# Efficient Feature Selection and Classification of Protein Sequence Data in Bioinformatics

**DOI:** 10.1155/2014/173869

**Published:** 2014-06-19

**Authors:** Muhammad Javed Iqbal, Ibrahima Faye, Brahim Belhaouari Samir, Abas Md Said

**Affiliations:** ^1^Computer and Information Sciences Department, Universiti Teknologi PETRONAS, Bandar Seri Iskandar, 31750 Tronoh, Perak, Malaysia; ^2^Fundamental and Applied Sciences Department, Universiti Teknologi PETRONAS, Bandar Seri Iskandar, 31750 Tronoh, Perak, Malaysia; ^3^College of Sciences, Alfaisal University, P.O. Box 50927, Riyadh 11533, Saudi Arabia

## Abstract

Bioinformatics has been an emerging area of research for the last three decades. The ultimate aims of bioinformatics were to store and manage the biological data, and develop and analyze computational tools to enhance their understanding. The size of data accumulated under various sequencing projects is increasing exponentially, which presents difficulties for the experimental methods. To reduce the gap between newly sequenced protein and proteins with known functions, many computational techniques involving classification and clustering algorithms were proposed in the past. The classification of protein sequences into existing superfamilies is helpful in predicting the structure and function of large amount of newly discovered proteins. The existing classification results are unsatisfactory due to a huge size of features obtained through various feature encoding methods. In this work, a statistical metric-based feature selection technique has been proposed in order to reduce the size of the extracted feature vector. The proposed method of protein classification shows significant improvement in terms of performance measure metrics: accuracy, sensitivity, specificity, recall, F-measure, and so forth.

## 1. Introduction

Bioinformatics has been an active area of research for the last three decades and is continuously gaining thoughtful attention from computer scientists and biologists research community. The objectives of bioinformatics were to store and manage the biological data and develop sophisticated computational tools that are helpful in the analysis and modeling [1]. The volume of data gathered in the Human Genome Project (HGP: 1990–2003) and various other successful sequencing projects is increasing exponentially, which raised many challenges for the research community [2]. The data generally consists of deoxyribonucleic acid (DNA), ribonucleic acid (RNA), and proteins. The most fundamental element of any living organism is proteins. It comprises 20 amino acids that carry out an important role in cell functions including nutrient transportation, metabolism regulation, and muscle building. A protein may adapt four different types of conformations due to some structural changes in order to perform functions inside the cell in the human body [3]. Every unknown protein needs annotation to know its structure and function, while the speed of the in vitro experiments is lessened quite a bit as more and more novel sequences are added constantly in the protein databases. However, the experimental methods are finding difficulties in annotating new proteins as they are very labor intensive and take a long time.

The homology-based approaches also have been utilized to predict the function of unannotated proteins by finding the sequence homology found between sequences in the databases. Two main categories of sequence homology-based approaches are alignment-based and alignment-free. Alignment-based models depend on single or multiple alignments to construct different types of models. Recently, techniques like basic local alignment search tool (BLAST), FAST-ALL (FASTA), and hidden Markov models (HMM) were the most reliably used alignment-based traditional methods for the analysis of both protein and DNA sequences. The results of protein BLAST show which segment or part of the protein sequence has more matches with the already available protein sequences in the database. BLAST uses the heuristic algorithm to measure the statistical significance of matched sequences in order to find similarity among them, while FASTA exploits local sequence alignment to find similar sequence using heuristic search in the database [4–8]. HMM is a probabilistic model or simple Bayesian model with hidden states [9, 10]. An HMM model is constructed for each family separately. The results of the aligned sequences of amino acid residues are generally represented as rows of a matrix. Generally, obtaining an efficient multiple alignment looks impossible when the sequences do not have enough similarity between them. Sequence alignment programs use a scoring matrix such as point accepted mutation (PAM) and BLOcks SUbstitution Matrix (BLOSUM) to generate a score for the alignment [11]. Some limitations of alignment-based approaches are [12] as follows.

- Alignment-based techniques undergo performance degradation on sequences having very weak or low similarity among them.
- Alignment-based techniques are heuristic in nature and thus are computationally expensive and take a long time on large datasets.
- Alignment-based techniques assume that contiguity is preserved within homologous segments, but this may not be accurate in genetic recombination.

The limitations of the alignment-based protein classification have been removed by the alignment-free classification techniques [12–18]. These techniques obtain different descriptors from each protein sequence (like the composition of amino acid, amino acid frequencies, and different chemical properties).

In the past, several machine learning approaches have been developed for the classification of protein sequences into functional or structural existing superfamilies [16, 19–22]. A superfamily is comprised of a set of proteins that possess sequence or structural homology. In a superfamily classification, an unlabeled protein sequence *Seq* may belong to any of the superfamily from a set of known superfamilies *F*_*i*_, *i* = 1,2,…, *m*. The computational techniques analyze whether the protein *Seq* belongs to any of the  *F*_*i*_, *i* = 1,2 …, *m* or whether it has no relation with any of them [17, 21]. It has been concluded from the previous literature [12–15, 17, 23–25] that similar protein sequences exhibit almost the same biological structure and function.

**Figure 1:**
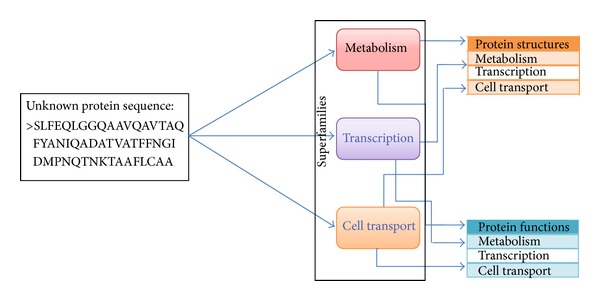
Prediction of the structure or function of an unknown protein.

Figure 1 explains the concept of the determination of the structure and function of any protein exclusively from the primary amino acid sequence. Moreover, Figure 1 demonstrates that, for a given unknown sequence, the classification technique investigates with which superfamily the new protein sequence belongs based on similarity with the existing sequences. In the figure, only three yeast sample superfamilies, namely, metabolism, transcription, and cell transport, were shown. The unknown sequence may belong to one of the three superfamilies based on the structure and function similarity.

The high dimensionality of biological data creates several crucial problems for the researchers during the implementation of machine learning based approaches during the analysis and modeling of extremely large amounts of sequence data. Many feature selection techniques have been introduced but still there is a need for a technique that can select statistically significant features for each protein sequence. The feature reduction would increase classification accuracy by removing the redundant or unnecessary features and also decrease the running time of classification algorithms. The automatic classification mechanism saves long time required for the experiments and the expenses of costly biological tests in laboratories. This research may be practically useful in drug discovery, drug design, and identification of genetic and proteomic diseases. A brief review of some recent techniques from the previous study is shown below.

Jeong et al. introduced a feature extraction method based on the position specific scoring matrix (PSSM) to extract features from a protein sequence [13]. The PSSM consisted of four components: position, probe, profile, and consensus. The authors defined four feature sets from the PSSMs. Feature set number 1 was obtained by dividing a protein sequence of any length into 20 equal sized blocks. Feature set number 2 considered domains having the same conservation ratio. Feature set number 3 extracted the physicochemical properties of the probing residues obtained from feature set number 2. Finally, feature set number 4 was proposed which consisted of all three feature sets. The total number of features investigated by this technique comprised a combination of four feature sets. Afterwards, the authors used four classifiers for the evaluation of classification technique: the naïve Bayesian (NB), support vector machine (SVM), decision tree (DT), and random forest (RF). Three yeast superfamilies (i.e., metabolism, transcription, and cellular transport) sequences were used as a training and test dataset. The maximum classification accuracy obtained was 72.5%. The accuracy was low due to a high misclassification rate. However, the accuracy could be further improved by extracting more relevant features from the protein sequence.

Mansoori et al. extracted features from a protein sequence using 2 grams and a 2-gram exchange group from the training and test data [12]. The distance-based feature ranking method was used for the selection of the best and most appropriate features. A SGERD-based classifier (steady state genetic algorithm for extracting fuzzy rules from data) was used to create fuzzy rules. Five superfamilies were considered in the experiments: globin, insulin, kinase, ras, and trypsin. These rules were then used for the classification of the protein sequences into superfamilies. The authors proposed a method that reduced the classification time from 79 to 51 minutes, while the classification accuracy was 96.45%. The time required for the classification could be further reduced and there would also be fewer chances that similar 2 grams would occur in unrelated sequences. Further improvement could also be made in the classification accuracy and the running time of the classification algorithm by the application of an appropriate feature selection technique.

Bandyopadhyay proposed a method that used a 1-gram technique for feature encoding [21]. The feature size was comprised of 20 amino acids. The extracted feature reflected the probability with which each amino acid occurred in any protein sequence. The authors proposed a variable-length fuzzy genetic clustering algorithm to find prototypes for each superfamily. For classification of protein sequences to relevant superfamilies, the nearest neighbor algorithm was employed. Three superfamilies, globin, ras, and trypsin, were utilized in the experiments. The classification accuracy obtained on the mentioned dataset was 81.3%. The classification accuracy can be enhanced using highly informative and more relevant features to describe a variable-length protein sequence.

In addition to the above works, in [26], the authors used different physicochemical properties to represent the features of a protein sequence. Only the distinguished and invariant features were used in the experiments. In the experiments, three superfamilies, such as esterase, lipase, and cytochrome, were investigated. The extracted features were given as input to the feed-forward, probabilistic neural network and radial basis function neural network. The probabilistic neural network showed accuracy of 90.6% on three superfamilies: esterase, lipase, and cytochrome. The classification accuracy might be increased by introducing a feature selection technique that has good discrimination power during classification.

Leslie et al. proposed a spectrum kernel to measure the sequence similarity between protein sequences [27]. The technique considered subsequences of *k* length amino acids (*k*-spectrum kernel) as a feature vector. The feature vector space obtained from the spectrum kernel was then passed to a support vector machine for classification of protein sequences into their relevant classes. The experiment was performed on SCOP superfamilies dataset that is comprised of 33 superfamilies. The experimental results were compared with SVM-T98, SVM-Fisher, and PSI-BLAST, which showed that SVM-Fisher produced better results as compared to other approaches. The performance of the technique could be further enhanced by the application of a suitable feature selection technique.

Caragea et al. investigated the feature hashing technique to map high dimensional features to low dimension using hash keys [28]. The high dimensional features were obtained using the traditional *k*-gram representation. The feature vector obtained after applying hash function stores frequency counts of each *k*-gram hashed together in the same hash key. With this technique, multiple features can be mapped to the same key. The size of the feature vector has been reduced from 2^22^ to 2^10^ without a major decrease in the classification accuracy. The proposed technique reduces the features' size and obtained classification accuracy of 82.83% using 1–4 gram sequence features in the classification of subcellular localization superfamilies, plant, and nonplant superfamilies datasets. Although the feature size was reduced, despite this, the feature size is still high, which needs to be reduced further more. The classification accuracy results could also be further increased using an optimized feature encoding and selection technique.

Yu et al. proposed a *k*-string dictionary technique to represent a protein sequence [29]. The value of “*k*” can be 1, 2,…,*k* amino acids. Repeated *k*-strings were considered only once. The frequency or probability of each *k*-string vector was observed during the experiments. Singular value decomposition (SVD) was then applied for the factorization of frequency/probability matrix, representing each protein sequence properly. Using the proposed technique the size of the feature vector was reduced. The experiment was performed on 290 simulated and real protein sequences of three families (PF03296, PF06924, and PF09455) from the Pfam database.

The review of existing protein sequence classification techniques demonstrates that many artificial intelligence, data mining, pattern recognition, and different statistical techniques have been proposed to find similarities or homology among the protein sequences from different superfamilies. However, the fast growth in the bioinspired sequence data has complicated the implementation of such techniques. The traditional approaches also find difficulties in the annotation of unknown sequence due to the noisy, incomplete, and high dimensions of the input sequence data. Thus, there is a need for a highly accurate and efficient feature subset selection system that can accurately classify the novel protein sequences into existing superfamilies and provides biologically useful information to the biologists in a very short amount of time.

In this paper, our objective is to overcome the limitations of high dimensional sequence data by introducing a statistical metric for the selection of discriminated or more informative features from a protein sequence. The proposed technique selects the most significant features which lead to improved classification results on various kinds of superfamilies sequences obtained from the publically known benchmark datasets. The incorporated technique is similar to alignment-free sequence classification methods and has significant advantages over the existing classification methods. Moreover, the proposed technique is very simple, fast, reliable, and robust and requires a very short training time.

The paper is organized as follows. In the Introduction, a brief description of bioinformatics and classification techniques from the literature has been presented. In Section 2, materials and methods which include the proposed methodology and techniques for feature encoding, selection, and classification are described.  Section 3 illustrates the experimental results obtained on the selected datasets and the comparisons of the proposed technique results with the already available best results. The results are discussed in Section 4 and the conclusion is presented in the final section.

## 2. Materials and Methods

**Figure 2:**
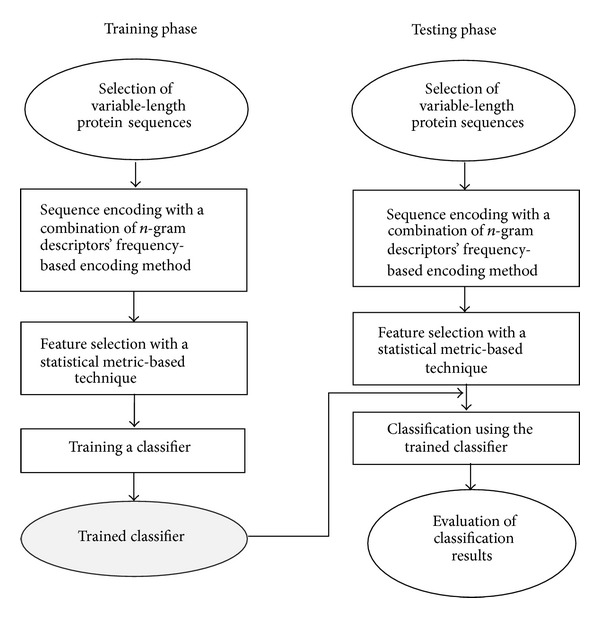
Phases of the proposed methodology.

Figure 2 presents the phases of the proposed methodology which have been involved during the classification of protein sequences into their particular superfamilies. Each phase performs some specific task during the classification process and has a significant importance. The methodology begins with the selection of training data and then proceeds with the sequence encoding and feature selection modules. After selection of statistically significant features, different classifiers have been used during the training. After a successful training, the system is tested on unknown data and classification performance of the proposed technique has been evaluated. The comprehensive detail of each phase is described in the next subsections.

### 2.1. Selection of Datasets from the Protein Database

The data used in the simulations was obtained from the UniProt knowledge database (UniProtKB) [30–33]. UniProt is a central repository to access comprehensive proteins sequences with functional information about various organisms and species. The database is comprised of two parts, which are SWISSProt (manually curated and reviewed sequences) and TrEMBL (automatically annotated and not reviewed sequences). The extracted sequences were nonredundant and comprised of nine different functionally important superfamilies. The details of the datasets used in each experiment are presented next in Tables 4, 5, and 6.

### 2.2. Training and Testing the System

The system was trained on the input data taken from the datasets mentioned in Tables 4, 5, and 6. The main purpose of the training is to minimize the error output produced by the system in comparison with the desired output. Furthermore, ten runs of the classification algorithm have been performed on the training and testing data. The training and testing phases involved different modules: encoding of the protein sequences, the feature subset selection, and implementation of the classification algorithm. After successful training of the system on the first 70% sequences, a classification model was built. The system was then tested on the protein sequences which were not used in the training phase (i.e., remaining 30% of the sequences).

### 2.3. Encoding of Protein Sequences

All protein sequences are represented by a combination of twenty amino acids. The representation of these protein sequences in the form of a minimum number of numeric features is an important problem in machine learning and bioinformatics. In this module of the methodology, the selected dataset is preprocessed before it is utilized in the training or testing phase. Selected datasets are then transformed into a feature vector space using a feature encoding method that extracts relative features from protein sequences. Sequence encoding methods greatly affect the quality and applicability of the machine learning techniques. In this paper, a sequence encoding method based on the combination of different n-gram (i.e., *n* = 1, 2, 3) descriptors is utilized for the extraction of valuable features from a protein sequence. The next subsection describes the details of the encoding method.

#### 2.3.1. Sequence Encoding by the Combination of n-Gram Descriptors' Frequency

An alignment-free sequence encoding technique based on the combination of different *n*-gram descriptors was employed during the classification process. A technique based on single amino acid frequency has been already used in a previous study [18]. This technique is identical to *k*-spectrum kernel or simple *n*-gram encoding method [17, 24, 27, 34].

By employing this encoding scheme, both the global and local features were extracted from a protein sequence. The protein descriptors of length 1, 2, 3,…, *n* amino acids could be constructed. In this work, we have investigated features of the lengths of 1, 2, and 3 amino acids. The *n*-gram descriptors with length bigger than 3 could be examined but this would result in the increase of the computational cost.

Below is a list of the twenty standard amino acids. These are used to specify protein sequences of any length for any gene:

(1) $$\begin{array}{c} X=\left\{\text{A},\text{C},\text{D},\text{E},\text{F},\text{G},\text{H},\text{I},\text{K},\text{L},\text{M},\text{N},\text{P},\text{Q},\text{R},\text{S},\text{T},\text{V},\text{W},\text{Y}\right\}, \end{array}$$

Let *X* ^I^, *i* = 1,2,…, *n* represent a set of protein descriptors of the length I amino acids. For example, *X* ^1^ = {A, C,…, Y}, *X* ^2^ = {AA, AC⁡,…, YY}.

The total number of protein descriptors of the length of 1, 2,…, *n* amino acids will be

(2) $$\begin{array}{c} N_{\text{Total}}=\text{Card}\;X^{1}+\text{Card}\;X^{2}+\cdots +\text{Card}\;X^{n}. \end{array}$$

where Card  *X* ^*i*^ represents the cardinal of *X* ^*i*^. Consider

(3) $$\begin{array}{c} N_{\text{Total}}=20+20^{2}+\cdots +20^{n}=\frac{20-20^{n-1}}{1-20}. \end{array}$$

For *n* = 3, the value of *N*_Total_ is 8420.

For example, suppose we have a protein sequence SAGKDNITLV; then the protein descriptors of the length 1 amino acid (i.e., *X* ^1^) are {S, A, G,…, V}. The protein descriptors of the length 2 amino acid (*X* ^2^) are {SA, AG, GK, KD,…, LV}. The protein descriptors of the length 3 amino acid (*X* ^3^) are {SAG, AGK, GKD …, TLV}. Similarly, the protein descriptors of the lengths 4, 5, up to *n* amino acids (*X* ^*n*^) can be attained.

The frequency of protein descriptors extracted from the five sample sequences is illustrated below in Tables 1, 2, and 3:

 Protein sequence-1  MKVLIFACM;
 Protein sequence-2  MKLCMKVL;
 Protein sequence-3  ACMKVLIFAC;
 Protein sequence-4  MKLIFACM;
 Protein sequence-5  CMKVIFACM.

In Table 1, the occurring frequency of each amino acid alphabet observed in each sequence is shown. For instance, the frequency of M in the first sequence is 0.22 which means that the occurrence probability of M in this sequence is approximately 22% and the frequency of K in the second sequence is 0.25, which shows that the occurrence probability of K in this sequence is approximately 25%. Similarly, the occurrence frequency/probability value of other amino acids is illustrated in the frequency table. The amino acids that do not appear in a sequence have a zero value. The contribution of such amino acids in a sequence is considered null.

Table 2 shows the occurrence frequency of amino acid descriptors of the size of two alphabets. In the table, the occurrence frequency of MK in the first sequence is 0.12. Similarly, the occurrence frequency of KV in the second sequence is also 0.14. Few protein descriptors having length of two amino acid alphabets were considered.

Table 3 shows the frequency of amino acids descriptors of the size of 3 alphabets. The occurrence frequency of MKV in the first sequence is 0.14 and the occurrence frequency of KVL in the second sequence is 0.16. Similarly, the occurrence frequency of the other amino acid descriptors could be determined.

When designing a machine learning technique for biological data, a feature vector space is constructed by extracting different features from the protein's primary sequence. To represent an unknown protein sequence, in this paper, a feature vector space based on the combination of *n*-gram protein descriptors' frequencies has been used. The technique got the frequency value of any feature for a sequence. The final feature vector space contained protein descriptors of lengths 1, 2, and 3 amino acids. The protein descriptors of length greater than 3 amino acids may be considered depending upon the complexity of data. This technique provides the evolutionary profile information about multiple sequences belonging to a particular superfamily. In the experiments, the data of different functional superfamilies, namely, metabolism, transcription, cell transport, esterase, lipase, cytochrome, globin, ras, and trypsin, were used. The frequency of the amino acid descriptors of the lengths of 1, 2, and 3 was calculated for each sequence for each superfamily.

### 2.4. Proposed Feature Selection Technique

From the above-described feature encoding method, each protein sequence is represented by a vector of 8420 features. Many features in the feature vector may have zero or empty values. This vector also contains several irrelevant or redundant features which provide no information about a protein sequence. These redundant features greatly affect the performance and running time of the sequence classification algorithms. In the proposed feature subset selection technique, the statistical significance of each feature of a superfamily from all other superfamilies is measured. The features that do not contribute in the representation of a sequence are removed from the original feature space; this will substantially reduce feature vectors' dimension. The proposed feature selection technique extracts different subsets of features from the original feature space and selects the best feature subset that shows maximum accuracy results. The subset of the best and relevant features was used to discriminate between different protein classes or superfamilies. The technique does not change the original representation of the features but only selects a subset of the best features from them. The processed data, after the feature selection, is used during the classification which drastically minimizes the running time of the classification algorithms. The classification model that uses this feature selection technique will certainly acquire 5% to 10% higher accuracy, and the computational complexity of these systems will also be lower. Both the supervised and unsupervised learning algorithms can successfully use this feature subset selection technique. The technique will greatly reduce the system training time and the chances of overfitting. The following steps are performed for the selection of the features capable of separating different superfamilies. The mathematical detail of this technique is described underneath.

Denoted by *X* ^*i*^ is the *i*th superfamily of sequences. *X*_*k*_^*i*^ represents the *k*th sequence of the superfamily *X* ^*i*^. *k* = 1, 2,…, *N*_*i*_, where *N*_*i*_ is the number of sequences in the *i*th superfamily. *X*_*k*_^*i*^(*j*), *j* = 1, 2,…, 8420  , is the feature vector representing the *k*th sequence of the *i*th superfamily. For each superfamily, the mean vector is calculated as follows:

(4) $$\begin{array}{c} {\overset{-}{X}}^{i}\left(j\right)=\frac{\sum_{k=1}^{N_{i}}X_{k}^{i}\left(j\right)}{N_{i}},\;j=1,2,\ldots ,8420. \end{array}$$

The variance of each superfamily is calculated as follows:

(5) Si2(j)=∑k=1Ni(X−i(j)−Xki(j))2Ni−1

For each pair of superfamilies (say *p* and *q*) a vector of distance is calculated using the metric defined below:

(6) vdp,q(j)=|Xp−(j)−Xq−(j)|(Sp2(j)/NTotal)+(Sq2(j)/NTotal)

Since, in this paper, 3 superfamilies were considered in each experiment, Equation (6) gave a matrix of 3 rows and *N*_Total_ columns. The 3 rows corresponded to all of the pairs of the superfamilies, namely, metabolism-transcription, metabolism-cell transport, and transcription-cell transport.

For each column, the minimum of the 3 distances was selected as the final metric, as in (7). Since the objective was to find the best features capable of discriminating the different superfamilies, the columns corresponding to the highest values of the final metric were selected. Consider

(7) vd(j)=Min⁡p≠q⁡{vdp,q(j)}.

The size of the reduced set of features may vary from few features/attributes to hundred features. In the experiments conducted in our work, the feature size was reduced from 8420 to a few features only (approximately 20 to 50), which represents a feature reduction of less than 1%. The above-described metric could be useful for different kinds of data classification problems.

## 3. Experimental Results

In the experiments, three different datasets were investigated, which are comprised of various functionally essential superfamilies from yeast and human genomes. The functional categories included in the experiments from the yeast genome were metabolism, transcription, cellular transport, and transport facilities, while from the human genome the functional categories were globin, trypsin, ras, esterase, lipase, cytochrome, and so forth, [13, 21, 26]. The sequences belonging to these families were downloaded from the UniProt knowledge base (UniProtKB) protein database. The protein sequences for each superfamily were chosen randomly.

After selection of relevant sequences in each dataset from the UniProt, the sequences were encoded using the combination of *n*-gram protein descriptors' frequency-based encoding method. Once all of the sequences had been encoded, the feature subset selection method was applied to reduce the size of a feature vector. A tenfold cross validation model was used in order to assess the results of the experiments. Furthermore, each fold's data was divided into two complementary subsets; in the first subset, 70% of the sequences were used for the training and the remaining 30% for the testing. The popular classification and learning algorithms, naïve Bayes, decision tree, random forest, neural network, and support vector machine, with their default settings have been applied on the dataset mentioned in Tables 4, 5, and 6. The detailed experimental result with best performing classifier is shown in the form of confusion matrices over each dataset.

The total number of confusion matrices on three different datasets using five classification algorithms was 15. The confusion matrices of only the best performing classifier on three datasets are shown in Tables 7, 8, and 9. Three important functional superfamilies belonging to yeast were used in the experiments in dataset 1. Yeast is a unicellular fungus, which is involved in the fermentation of sugar, wheat, and so forth. It is also widely used in vitamin supplements. In dataset 2, the exclusive sequences of three superfamilies, globin, ras, and trypsin, were used for training and testing. Dataset 3 contained sequences which belong to 3 superfamilies: esterase, lipase, and cytochrome.

The rows' sum of confusion matrices shows actual sequences and the columns' sum represents the number of predicted sequences in each superfamily. The performance of the proposed technique has been validated on different datasets using performance measure metrics like true positive rate (TPR), false positive rate (FPR), specificity, sensitivity, recall, F-measure, and Mathews correlation coefficient (MCC) [13, 35]. It was observed that the least numbers of sequences were misclassified in our experiments. Only the neural network classifier offered a better performance as compared to the other classifiers in terms of accuracy, specificity, sensitivity, F-measure, and recall. The true positives rate and true negatives rate were high with the neural network classifier. The detailed discussion and empirical comparison of the performance measure metrics of classification algorithm on each dataset are shown in the following section.

## 4. Discussions

**Figure 3:**
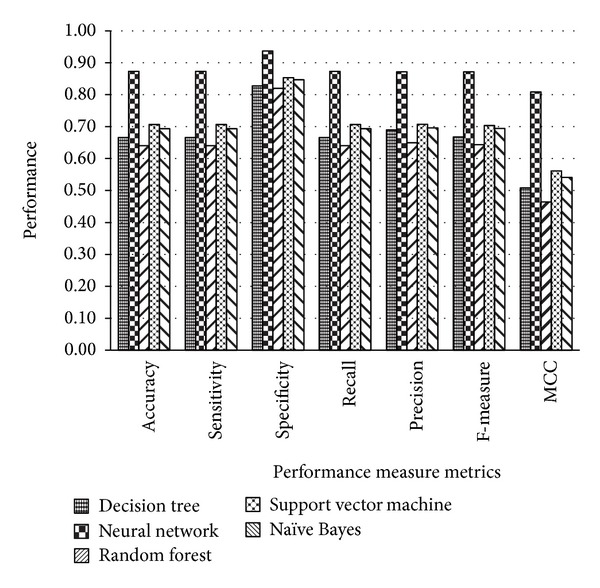
Comparison of the performance measure metrics using dataset 1.

**Figure 4:**
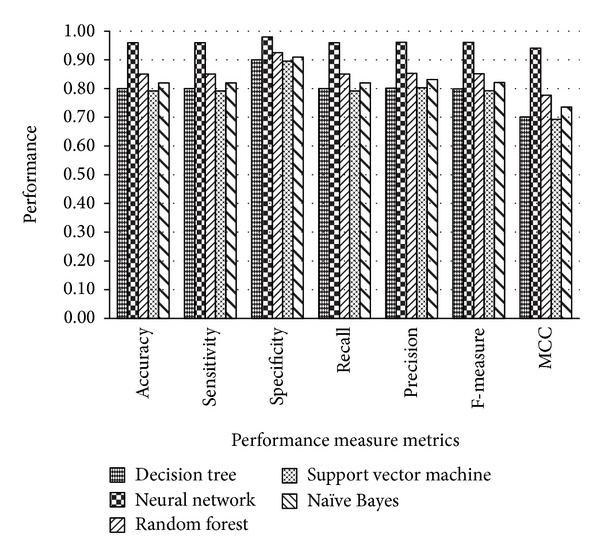
Comparison of the performance measure metrics using dataset 2.

**Figure 5:**
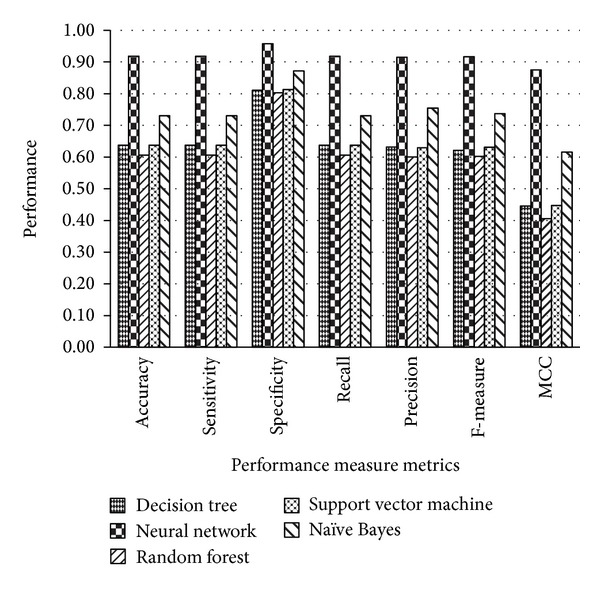
Comparison of the performance measure metrics using dataset 3.

Since precisely predicting the function or structure of a novel protein from a protein sequence is a significant problem in machine learning and bioinformatics research community, the exact knowledge about the structure and function of proteins provides a way to analyze and model protein sequences and is also helpful in the treatment of numerous diseases. This will also be useful in the design and discovery of new drugs for many diseases. In this study, nine functionally important superfamilies of protein sequences were taken into account; these are very essential to perform various critical functions. The variable-length protein sequences were chosen randomly from the benchmark UniProt database. The protein sequences in the chosen superfamilies are relatively long and have very low sequence similarity among them; therefore, it was very difficult to classify them into existing superfamilies with high accuracy using previous approaches. We have utilized a combination of *n*-gram protein descriptors' frequency-based features encoding to represent a protein in the form of a fixed-length feature vector. The statistical metric was employed for the selection of informative features which significantly reduced the size of the feature vector. The classification results using popular classification algorithms on each dataset have been shown in the next section. Figures 3, 4, and 5 showed the graphical representation of the performance measure metrics obtained on each dataset using different classification algorithms. In all of the graphs, the *x*-axis contains the performance measure metrics that we have measured in the experiments and on the *y*-axis; the values of the metrics obtained with each classifier are shown. The analysis of the graphs in Figures 3, 4, and 5 indicates that the neural network classifier on all three datasets shows improved classification accuracy, specificity, sensitivity, precision, recall, F-measure, and MCC. The trends of the improvements found in the performance measure metrics were very similar on the three datasets. The architecture of neural network used in our experiments was comprised of default parameters with 10 hidden neurons and 3 output layers.

**Figure 6:**
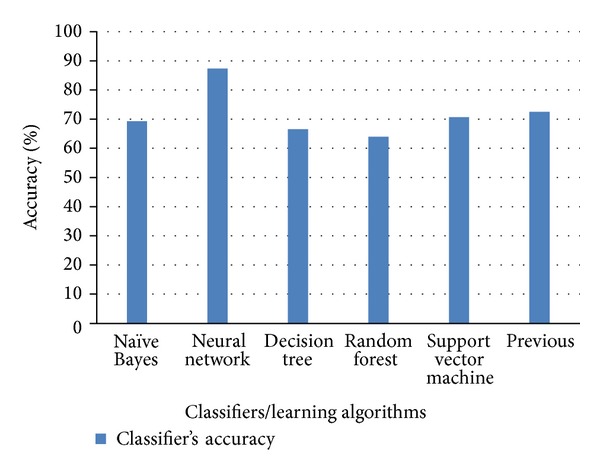
Comparison of each classifier's classification accuracy using dataset 1.

**Figure 7:**
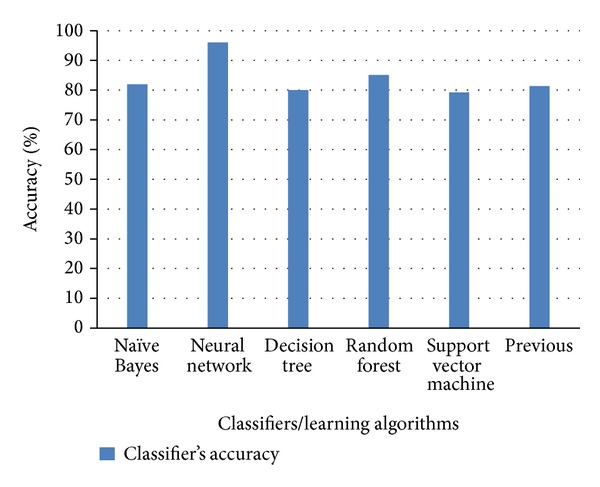
Comparison of each classifier's classification accuracy using dataset 2.

**Figure 8:**
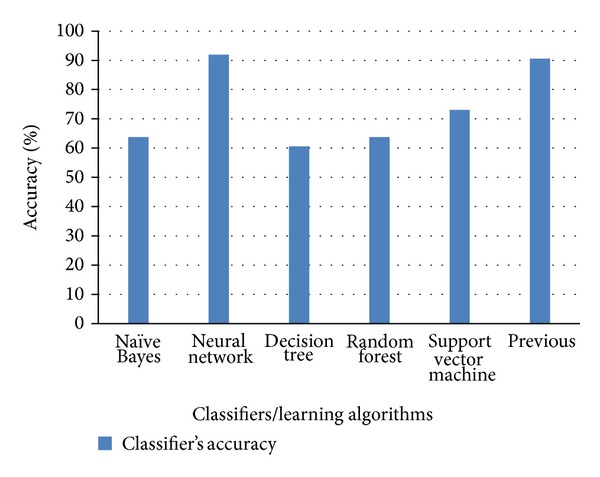
Comparison of each classifier's classification accuracy using dataset 3.

**Figure 9:**
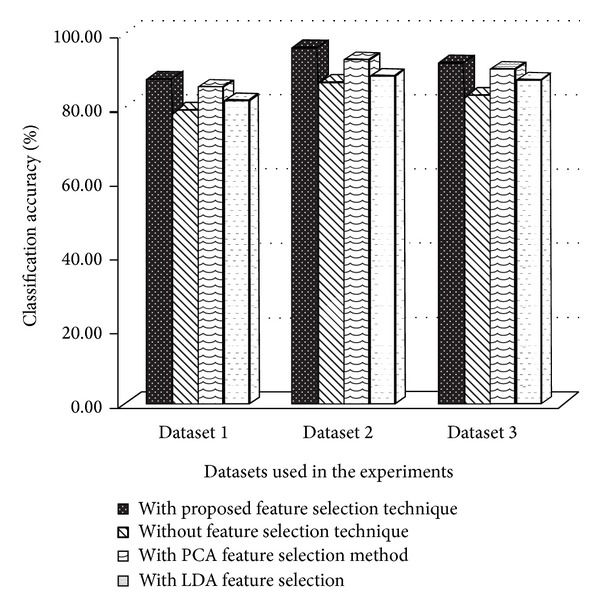
Significance of the feature subset selection technique on classification accuracy of three datasets.

**Figure 10:**
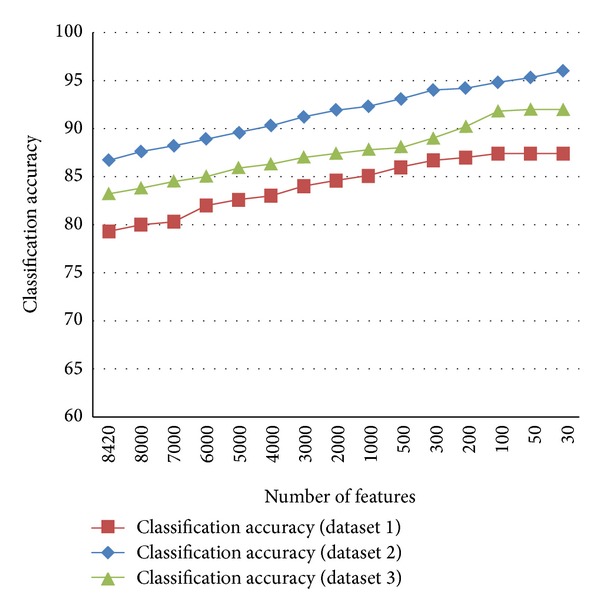
Number of features versus classification accuracy on three datasets.

Figures 6, 7, and 8 show the comparison of existing and proposed classification accuracy results obtained on different datasets using the proposed sequence encoding and feature subset selection techniques. Without using the feature subset selection technique, the feature size would be bigger and this would ultimately decrease the classification accuracy and more computational cost would be required.  Figure 6 shows the comparison of the accuracy of the proposed method with the previously available classification methods. The maximum average accuracy obtained on dataset 1 using the neural network was 87%, which was 15% more than the previous study [13]. Similarly, the classification accuracy using dataset 2 is presented in Figure 2. The achieved accuracy was 96% which was approximately 14% more than the previous [21].  Figure 8 illustrates the comparison of the achieved classification accuracy (i.e., 92%) on dataset 2 with the accuracy of the previous study [26]. The employed feature encoding was also evaluated using all 8420 features. The plot in Figure 9 shows the significance of the feature subset selection on the classification accuracy of all of the datasets involved in the experiments. The experiments were performed using all 8420 features and reduced the number of features selected with the proposed feature selection technique. The plot in Figure 9 indicates that, without using the feature subset selection, the classification accuracy was approximately 5% to 10% lower. This was due to the fact that there may have been some irrelevant features that contained no information about any protein sequence.  Figure 10 illustrates the effect of feature selection on the classification accuracy. The graph demonstrates that an increase in number of features may decrease the classification accuracy. Thus the correct use of the proposed feature selection technique can be helpful for feature/dimensionality reduction problems. Although previously existing feature selection techniques such as principal component analysis (PCA), linear discriminant analysis (LDA), and probabilistic latent semantic analysis (PLSA) have been applied to select the most significant and relevant features [36–38], however, these techniques have shown average accuracy results and were also found to be computationally expensive for high dimensional data obtained from different representations of protein sequences. However, from our experiments, the impact of feature selection could be observed.

The empirical analysis of the results shows that the statistical metric-based feature selection technique extracted the more relevant and informative features from a variable-length protein sequence than the PSSM-based technique [13]. The proposed technique explores dependencies between the features in a subset and classes. The PSSM-based features are constructed using sequence alignment, which also show performance degradation on the protein sequences of different lengths. The neural network classifier in all of the experiments consistently showed promising results as compared to the other classifiers. Although the accuracy obtained with different datasets was different, the analysis of the results indicated that the classification accuracy primarily depends on the complexity of the amino acid sequences, the length of the individual protein sequence, and the size of the training and test data. The combine use of the suggested sequence encoding and feature subset selection techniques substantially increase the classification accuracy by minimizing false alarm rate. The sensitivity and specificity results of the overall classification process are highly appreciable. The proposed technique of protein sequence classification would be very helpful in the modeling and analysis of different kinds of biological data and it might be a good addition to the existing protein sequence classification techniques.

## 5. Conclusions

In this paper, a statistical metric-based feature subset selection technique has been introduced for the selection of discriminant and invariant features from a protein sequence. During the sequence encoding process, each protein sequence was represented with *n*-gram descriptors' frequency. In the experiments, the descriptors' sizes up to 3 amino acids were considered. The statistical metric discards the irrelevant or redundant features and represents the protein sequences with a minimum number of statistically discriminant features. The proposed feature subset selection technique uses a threshold to select the highly informative and important features. The results of the technique were validated through the well-recognized classification/learning algorithms. The protein sequences of three different datasets have been effectively classified into relevant superfamilies with substantially high classification accuracy. The introduced classification method is alignment-free, simple, fast, and reliable. This technique of feature selection and classification would be useful in machine learning and bioinformatics in reducing the high dimensionality of data during the prediction of the structure or function of unknown protein sequences. In the future, the proposed technique can be extended to other areas of pattern recognition like the classification of different kinds of proteomics and genetic diseases.

## Figures and Tables

**Table 1:** The frequency of *X* ^1^ (single letter amino acid descriptors) for each sequence.

Sequence number	M	K	V	L	I	F	A	C
1	0.22	0.11	0.11	0.11	0.11	0.11	0.11	0.11
2	0.25	0.25	0.13	0.25	0.00	0.00	0.00	0.13
3	0.10	0.10	0.10	0.10	0.10	0.10	0.20	0.20
4	0.25	0.13	0.00	0.13	0.13	0.13	0.13	0.13
5	0.20	0.10	0.10	0.10	0.10	0.10	0.10	0.20

**Table 2:** The frequency of *X* ^2^ (two letter amino acids descriptors) for each sequence.

Sequence number	MK	KV	VL	LI	IF	AC	CM	FA	KL
1	0.12	0.12	0.12	0.12	0.12	0.12	0.12	0.12	0.00
2	0.28	0.14	0.14	0.00	0.00	0.00	0.14	0.00	0.14
3	0.10	0.10	0.10	0.10	0.10	0.22	0.10	0.10	0.00
4	0.10	0.00	0.00	0.14	0.14	0.14	0.14	0.14	0.14
5	0.10	0.10	0.10	0.10	0.10	0.10	0.22	0.10	0.00

**Table 3:** The frequency of *X* ^3^ (three letter amino acids descriptors) for each sequence.

Sequence number	MKV	KVL	VLI	LIF	ACM	IFA	MKL
1	0.14	0.14	0.14	0.14	0.14	0.14	0.00
2	0.16	0.16	0.00	0.00	0.00	0.00	0.16
3	0.12	0.10	0.10	0.10	0.10	0.10	0.00
4	0.00	0.00	0.00	0.16	0.16	0.16	0.16
5	0.12	0.12	0.12	0.12	0.12	0.12	0.00

**Table 4:** Details of dataset 1 used in the experiments.

Superfamily name	Number of sequences
Metabolism	750
Transcription	520
Cellular transport, transport facilities	560
Total sequences	**1830**

**Table 5:** Details of dataset 2 used in the experiments.

Superfamily name	Number of sequences
Globin	250
Trypsin	250
Ras	250
Total sequences	**750**

**Table 6:** Details of dataset 3 used in the experiments.

Superfamily name	Number of sequences
Esterase	190
Lipase	150
Cytochrome	140
Total sequences	**480**

**Table 7:** Confusion matrix obtained using neural network classifier on dataset 1.

Data*∖*results	Metabolism	Transcription	Cellular transport
Metabolism	201	9	15
Transcription	6	142	8
Cellular transport	17	14	137

**Table 8:** Confusion matrix using neural network classifier on dataset 2.

Data*∖*results	Globin	Ras	Trypsin
Globin	73	1	1
Ras	2	71	2
Trypsin	2	1	72

**Table 9:** Confusion matrix obtained using neural network classifier on dataset 3.

Data*∖*results	Esterase	Lipase	Cytochrome
Esterase	51	2	4
Lipase	2	42	1
Cytochrome	2	1	39
